# AI-driven optimization in cloud computing: a systematic review of cost, resource management, and security

**DOI:** 10.3389/frai.2026.1750992

**Published:** 2026-04-30

**Authors:** Ronaldy Solano Ito López, Romel Gutierrez Oscata, Ángel Rosendo Condori-Coaquira, Ronny Ivan Gonzales Medina, Javier Linkolk López-Gonzales, Esteban Tocto-Cano

**Affiliations:** 1 Escuela Profesional de Ingeniería de Sistemas, Facultad de Ingenieria y Arquitectura, Universidad Peruana Unión, Juliaca, Peru; 2 Facultad de Ciencias e Ingenierías Físicas y Formales, Universidad Católica de Santa María, Arequipa, Peru; 3 Escuela de Posgrado, Universidad Peruana Unión, Lima, Peru

**Keywords:** artificial intelligence, cloud computing, cost optimization, deep learning, machine learning, resource management, security, systematic review

## Abstract

Cloud computing environments face persistent structural challenges in cost control, dynamic resource allocation, and security risk management, which traditional infrastructure approaches fail to address adequately. This systematic literature review aimed to synthesize empirical evidence on the application of artificial intelligence (AI) and machine learning (ML) models for cost optimisation, resource management, and security enhancement in cloud computing environments. Following the PRISMA 2020 guidelines and the Kitchenham–Charters methodology, a structured search was conducted across IEEE Xplore, Web of Science, ScienceDirect, and the ACM Digital Library, covering the period 2020–2025. From an initial pool of 216 records, 18 primary studies were selected after applying the PICOC framework, predefined inclusion and exclusion criteria, and a dual-reviewer quality assessment process yielding substantial inter-rater agreement (Cohen's κ = 0.86). The synthesized evidence demonstrates that predictive provisioning systems and intelligent load-balancing mechanisms reduce operational costs by up to 85%, metaheuristic algorithms such as the Whale Optimization Algorithm and Particle Swarm Optimization improve energy efficiency by 30%–40% and increase resource utilization by up to 80%, and deep learning–based intrusion detection systems achieve accuracy levels exceeding 92%. These findings confirm that AI constitutes a structural mechanism for strengthening economic efficiency, operational resilience, and the sustainability of cloud infrastructures. However, heterogeneity in simulation environments, limited validation in production-scale deployments, and insufficient coverage of virtual machine migration dynamics represent critical gaps requiring standardized benchmarking frameworks and empirical validation in hybrid and multicloud architectures. A quantitative synthesis (Table 1) reveals that metaheuristic algorithms achieve 30%–40% cost and energy efficiency improvements, while ensemble deep learning approaches attain >97% security threat detection rates.

## 1 Introduction

Cloud computing constitutes the backbone of contemporary digital infrastructure by delivering scalability, elasticity, and cost efficiency that exceed those of traditional on-premise architectures (Ramasamy and Gnanamurthy, 2020). The integration of Artificial Intelligence (AI), Machine Learning (ML), and Deep Learning (DL) further transforms cloud environments by enabling predictive analytics, intelligent automation, workload optimization, and real-time decision-making across distributed platforms (Wang, 2022). Despite rapid adoption, organizations continue to encounter structural challenges that constrain performance and sustainability. In particular, dynamic pay-per-use pricing models complicate cost control, fluctuating workloads strain resource allocation mechanisms, and increasingly sophisticated cyber threats expose vulnerabilities, especially in data-sensitive sectors such as healthcare and finance. These persistent constraints necessitate adaptive, data-driven optimization strategies capable of addressing multidimensional and interdependent operational objectives.

Existing research on AI-driven cloud optimization remains fragmented, as prior studies typically examine isolated dimensions such as performance enhancement, anomaly detection, or cost reduction without integrating these perspectives into a unified analytical framework. This compartmentalization limits the systematic understanding of AI's collective contribution to efficiency, security, and sustainability within cloud ecosystems. Moreover, previous reviews rarely provide comparative analyses of diverse AI paradigms, including ML, DL, and reinforcement learning, across multiple optimization domains. Current research also exhibits methodological and contextual limitations, including the absence of standardized datasets for reproducible evaluation, limited validation in hybrid and multicloud environments, insufficient large-scale deployment testing, and inadequate examination of the ethical, regulatory, and privacy implications associated with AI-enabled cloud systems (Gaur et al., 2024; Sakthidevi et al., 2023; Lim et al., 2018). Addressing these deficiencies requires a rigorous synthesis that consolidates empirical evidence while critically evaluating methodological robustness and practical applicability.

This systematic literature review addresses these gaps by synthesizing advances published between 2020 and 2025 using PRISMA 2020 guidelines and the Kitchenham–Charters methodology. The review systematically evaluates AI techniques across three interrelated optimization domains cost reduction, resource management, and security enhancement and provides evidence-based insights into their effectiveness, comparative performance, limitations, and future potential within complex cloud environments.

This study makes six principal contributions.

First, it delivers a comprehensive synthesis that integrates AI's impact on cost optimization, resource management, and security in cloud computing during 2020–2025.

Second, it provides quantitative evidence derived from 18 high-quality studies, demonstrating measurable improvements, including cost reductions of up to 85%, security detection accuracy exceeding 92%, and energy efficiency gains approaching 40%.

Third, it ensures methodological rigor through the application of the Preferred Reporting Items for Systematic Reviews and Meta-Analyses (2020) guidelines, the PICOC framework, and dual-reviewer quality assessment with substantial inter-rater agreement (Cohen's κ = 0.86), thereby enhancing reproducibility and minimizing bias.

Fourth, it offers a comparative analysis of AI techniques, including deep learning, reinforcement learning, and metaheuristic optimization algorithms, across distinct cloud optimization objectives.

Fifth, it systematically identifies research gaps related to dataset standardization, hybrid and multicloud validation, and large-scale production deployment.

Finally, it proposes a forward-looking research agenda that prioritizes federated learning, edge-cloud collaboration, explainable AI, and generative AI to advance autonomous cloud management.

This review generates both practical and theoretical benefits. For cloud service providers, the synthesized evidence clarifies how AI-driven optimization strategies can substantially reduce operational costs while improving security and resource efficiency. For researchers, the study consolidates dispersed knowledge, highlights critical methodological and empirical gaps, and delineates promising directions for next-generation cloud systems. Policymakers and industry practitioners also gain an evidence-based understanding of AI's transformative capacity and its associated governance and deployment challenges within evolving cloud ecosystems.

The remainder of this paper is organized as follows. Section 2 reviews related work on AI in cloud computing. Section 3 describes the systematic review methodology, including the search strategy, selection criteria, and quality assessment procedures. Section 4 presents the results across the cost optimization, resource management, and security domains. Section 5 discusses the findings, limitations, and broader implications. Section 6 concludes the paper and outlines future research directions.

## 2 Related works

Cloud computing has consolidated itself as a central paradigm of contemporary digital infrastructure through the systematic integration of Artificial Intelligence (AI) and Machine Learning (ML) to optimize resources, strengthen security, and enhance infrastructure management. Specialized literature demonstrates that AI-driven approaches effectively address the structural challenges associated with dynamic resource allocation, energy efficiency, and quality of service (QoS) assurance in highly heterogeneous environments. Within this framework, state of the art AI-based cloud optimization is organized around infrastructure management, demand prediction, distributed architectures, security, and emerging technologies.

Resource optimization constitutes the operational core of modern cloud systems, as it determines infrastructure efficiency, scalability, and sustainability. Numerous studies have proposed orchestration and adaptive allocation mechanisms for heterogeneous environments. (Khan et al. 2019) introduced H2, a heterogeneity-aware orchestrator that manages resources according to their specific properties; in contrast, (Zakarya et al. 2022) proposed CoLocateMe to optimize virtual machine placement and consolidation in heterogeneous Infrastructure-as-a-Service (IaaS) environments, achieving substantial improvements in utilization and energy efficiency. Similarly, ReAssigner (Khan et al., 2019) enhanced the adaptability of existing schedulers through physical role preassignment and virtual cluster formation, and (Abdullah and Surputheen 2024) demonstrated that evolution strategies outperformed traditional genetic algorithms in terms of makespan, throughput, and utilization. Complementarily, (Zakarya et al. 2024a), proposed BackFillMe (Zakarya et al., 2025), an energy- and performance-efficient virtual machine scheduler for IaaS datacenters that reduces energy consumption while maintaining high throughput, and FollowMe@LS (Ali et al., 2021), which addresses resource management in geographically distributed heterogeneous datacenters by accounting for electricity price and source variability. Collectively, these approaches confirm that AI-driven intelligent orchestration consistently improves performance and efficiency across cloud ecosystems.

VM migration is a critical mechanism for sustaining energy efficiency and performance in dynamic cloud environments. (Zakarya 2018) presented an extended energy-aware cost recovery framework for VM migration that explicitly models the tradeoff between migration overhead and subsequent energy savings, demonstrating that naive migration policies can negate efficiency gains. This line of work is complemented by research on cloud simulation fidelity: (Zakarya and Gillam 2019) quantified the impact of resource heterogeneity modeling on simulation accuracy, showing that ignoring heterogeneity introduces significant prediction errors. For large-scale scenarios, PerficientCloudSim (Zakarya et al., 2021) extends CloudSim to support heterogeneous workloads at scale, enabling more realistic benchmarking of the algorithms reviewed in this study.

Bio-inspired metaheuristics further expand the methodological repertoire for energy and performance optimization. (Rawat et al. 2020) develop a bio-inspired neural model that reduces energy consumption without compromising performance, and (Goyal et al. 2021) applied the Whale Optimization Algorithm to achieve energy-efficient resource allocation. Complementarily, (Zhou et al. 2021) incorporated clustering techniques with enhanced differential evolution to expand the solution space in cloud optimization problems. These findings indicate that integrating metaheuristic optimization with predictive modeling strengthens the dynamic management of complex infrastructures. Broader sustainability considerations were addressed by (Zakarya 2018), whose systematic review of sustainable computing models across datacenters provides a comprehensive taxonomy of energy-aware techniques directly relevant to the optimization strategies examined in this review.

Accurate demand forecasting is critical to ensuring QoS and operational efficiency in cloud environments. (Nawrocki and Osypanka 2021) develop ML-based predictive models capable of identifying usage patterns with high accuracy, thereby enabling proactive allocation and reducing resource waste. Complementarily, (Rawat et al. 2020) identified structural challenges related to allocation, scalability, and energy management, underscoring the need for integrated multi-objective approaches. The literature converges on the conclusion that data-driven anticipation represents a key mechanism for balancing performance, cost, and sustainability.

Distributed and hybrid architectures require scheduling strategies that integrate temporal constraints, security requirements, and low-latency conditions. In hybrid cloud environments, (Abdi et al. 2025) propose a security-aware scheduling scheme under deadline constraints. In the context of 5G networks, (Zheng et al. 2020) designed a hierarchical distributed architecture that optimized allocation and scheduling under stringent latency requirements. Moreover, (Arul et al. 2023) integrated Internet of Things (IoT) and edge computing within vehicle ad hoc networks (VANETs) for intelligent energy management. In the emerging domain of connected and autonomous vehicles (CAVs), ApMove (Zakarya et al., 2024a) proposes a service migration technique that maintains application continuity during vehicle mobility, representing a critical advancement at the convergence of cloud, edge, and IoT ecosystems.

The detection and mitigation of Distributed Denial-of-Service (DDoS) attacks constitutes a priority research line in AI-based cloud security. (Kushwah and Ranga 2021) develop an optimized Extreme Learning Machine (ELM) model for real-time detection, while (Sanjalawe and Althobaiti 2023) combine metaheuristic feature selection with CNN-LSTM architectures, achieving detection rates above 97%. Furthermore, (Mayuranathan et al. 2022) integrate hybrid optimization techniques with deep learning in advanced Deep K-Nearest Neighbors (DKNN) systems, and (Zakarya et al. 2025) consolidated the integration of big data analytics and deep learning to reinforce cloud security.

Table 1 summarizes the principal studies related to this research, including the present study's position relative to existing works across key dimensions: VM migration, energy consumption, geographically distributed IaaS, validation approach, and optimization type. Gaps in prior reviews—particularly the absence of VM migration modeling, limited treatment of geo-distributed IaaS, and missing coverage of sustainability frameworks—are explicitly addressed by the contributions synthesized in this review.

**Table 1:** Quantitative synthesis: AI model type × optimization area × performance metrics.

Optimization area	AI/ML model type	N	Metric type	Performance range	Best performer(s)	Notes
**Cost reduction**	WOA	2	Cost red. (%)	30%–40%	(Goyal et al., 2021)	Energy-aware allocation
	Algorithm-driven (unspecified)	1	Operating cost red. (%)	Up to 85%	(Golightly et al., 2022)	Conference paper; limited validation
	Differential evolution + clustering	1	Cost savings	Not quantified	(Zhou et al., 2021)	Indirect via resource efficiency
	Geo-distributed resource mgmt	1	cost red. (%)	>30%	(Nawrocki and Osypanka, 2021)	Azure ML studio
	ML predictive models	1	Operating cost red. (%)	>30%	(Nawrocki and Osypanka, 2021)	Azure ML studio
	**Summary**	**6**	Cost Red. (%)	**30%–85%**	(Golightly et al., 2022)^*^	^*^Outlier; median ≈30%–40%
**Energy efficiency**	WOA	2	Energy Eff. Improv. (%)	30%–40%	(Goyal et al., 2021)	Consistent across studies
	Bio-inspired neural models	1	Energy Eff. Improv. (%)	30%–40%	(Rawat et al., 2020)	Cited in discussion
	Energy-aware VM migration	1	Energy Savings (%)	Not quantified	(Abdi et al., 2025)	Qualitative improvement
	Edge-Cloud ML (Microgrid)	1	Energy Eff.	Not quantified	(Arul et al., 2023)	Focus on QoS
	**Summary**	**5**	Energy Eff. (%)	**30%–40%**	(Goyal et al., 2021)	Convergent evidence
**Resource allocation**	Differential evolution + clustering	1	Resource Eff. (%)	Up to 80%	(Zhou et al., 2021)	Load balancing focus
	Evolution strategies	1	Makespan & Throughput	Superior to GA	(Abdullah and Surputheen, 2024)	vs. genetic algorithms
	ML Predictive Models	1	Resource Util. Improv. (%)	>30%	(Nawrocki and Osypanka, 2021)	Demand prediction
	Q-Learning (5G Networks)	1	Resource optimization	Not quantified	(Zheng et al., 2020)	5G cloud-edge
	Security-aware Scheduling	1	Makespan & Cost	Not quantified	(Abdi et al., 2025)	Hybrid cloud
	**Summary**	**5**	Resource Eff. (%)	**30%–80%**	(Zhou et al., 2021)	Wide range; heterogeneous metrics
**Security & IDS**	Deep neural networks (ANN)	1	Detection Rate (%)	>90%	(Hasimi et al., 2024)	Cloud security focus
	Hybrid deep learning (DKNN)	1	Detection accuracy (%)	>92%	(Hasimi et al., 2024)	CNN, R-CNN comparison
	CNN-LSTM + Metaheuristic	1	Detection rate (%)	>97%	(Sanjalawe and Althobaiti, 2023)	DDoS; ensemble feature selection
	Hybrid CNN-LSTM	1	False positive red. (%)	Up to 25%	(Sanjalawe and Althobaiti, 2023)	Attack pattern detection
	Optimized extreme learning machine	1	Detection performance	Not quantified	(Kushwah and Ranga, 2021)	DDoS in cloud
	ML + Big data analytics	1	Threat detection	Not quantified	(Mohammad and Pradhan, 2021)	Real-time detection
	Hybrid deep learning (DKNN)	1	Intrusion detection	Not quantified	(Mayuranathan et al., 2022)	Cloud environment
	**Summary**	**7**	Detection rate/acc. (%)	**90%–97%**	(Sanjalawe and Althobaiti, 2023)	38.9% of total studies

**Table 2:** Comparative summary of related works.

Study	Contribution/ focus	VM Migr.	Energy consump.	Geo-Dist. IaaS	Validation	Optim. type
(Khan et al. 2019)	Heterogeneity-aware resource orchestration	No	Partial	Yes	Experimental	Resource allocation
(Zakarya et al. 2022)	VM placement & consolidation in IaaS	Yes	Yes	Yes	Baseline comparison	Energy + utilization
(Abdullah and Surputheen 2024)	Scheduling via evolution strategies	No	No	No	vs. GA	Makespan, throughput
(Rawat et al. 2020)	Bio-inspired ANN for energy optimization	No	Yes	No	CloudSim	Energy efficiency
(Goyal et al. 2021)	WOA for energy-resource allocation	No	Yes	No	Cloud analyst	Energy + resource use
(Zhang et al. 2008)	Predictive energy-aware scheduling	No	Yes	No	Dynamic workload sim.	Scheduling, energy
(Abdi et al. 2025)	Security-aware scheduling in hybrid cloud	No	No	Yes	CPLEX/OPL	Makespan, cost
(Zheng et al. 2020)	Hierarchical arch. for 5G cloud-edge	No	No	Yes	Q-learning emulation	Latency, allocation
(Kushwah and Ranga 2021)	ELM for DDoS detection	No	No	No	MATLAB, NSL-KDD	Detection accuracy
(Sanjalawe and Althobaiti 2023)	CNN-LSTM + metaheuristic for DDoS	No	No	No	Python/Keras, CICIDS	Detection, FP reduction
(Zakarya et al. 2025)	VM scheduler for energy + perf. in IaaS	Yes	Yes	Yes	CloudSim	Energy, makespan
(Ali et al. 2021)	Geo-distributed datacenter resource mgmt.	Yes	Yes	Yes	Simulation	Cost, energy, QoS
(Zakarya 2018)	Energy-aware cost recovery for VM migration	Yes	Yes	Yes	CloudSim	Migration cost, energy
(Zakarya and Gillam 2019)	Cloud simulation accuracy with heterogeneity	No	Yes	Yes	Custom simulation	Accuracy, fidelity
(Zakarya et al. 2021)	Large-scale heterogeneous cloud simulation	No	Yes	Yes	PerficientCloudSim	Scalability, performance
(Zakarya et al. 2024a)	Service migration for CAVs in IoT	Yes	Yes	Yes	Sim. + real traces	Migration latency, QoS
(Zakarya et al. 2024b)	Review of sustainable datacenter models	Yes	Yes	Yes	Systematic review	Energy, sustainability
**This Study**	SLR: AI for cost, resource mgmt. & security	Addressed	Yes	Yes	PRISMA/PICOC (18 studies)	Cost, energy, security

## 3 Methodology

This study adopts a systematic literature review (SLR) approach to identify, analyze, and synthesize the most relevant scientific evidence on the integration of artificial intelligence (AI) in cloud computing environments. Unlike narrative or unstructured reviews, the SLR is based on an explicit, transparent, and reproducible protocol, enhancing the reliability of results and minimizing bias.

To conduct the review, the methodological guidelines proposed by (Kitchenham and Charters 2007), which are widely accepted in software engineering and computer science. These guidelines define standardized procedures, from the formulation of research questions to the systematic analysis of evidence, including the construction of search chains and the selection of studies.

### 3.1 Protocol structure

The protocol was structured as follows:

1. Defining research questions and objectives using the PICOC framework (Population, Intervention, Comparison, Outcome, and Context).
2. Selection of search terms in high-impact databases, such as IEEE Xplore, Web of Science, ScienceDirect, and ACM Digital Library.
3. Selection of studies based on the inclusion and exclusion criteria to ensure relevance, timeliness, and methodological quality.
4. Manual screening of titles, abstracts, and keywords.
5. Assessment of the quality of the selected studies based on a standardized scale.
6. Data extraction using a systematic form for subsequent qualitative and quantitative synthesis.

The search time frame was set between 2020 and 2025 to ensure that the results capture the most recent and relevant research. English was prioritized, as it is the most widely used language in high-impact publications in engineering and computer science.

After applying the eligibility and quality criteria, 18 primary studies were selected from a total of 216 articles collected and analyzed. These studies underwent thematic and comparative analyses to, identify patterns, trends, strengths, and limitations in the use of AI for cost optimization, security, and resource management in cloud environments.

### 3.2 Questions and objectives

The objective of this review is to identify studies employing artificial intelligence (AI) and machine learning (ML) models for cost optimization and efficient resource utilization in cloud computing environments. To operationalize this objective, a set of research questions (RQs) was defined, as summarized in Table 3.

**Table 3:** Research questions (RQs) and corresponding objectives.

ID	Research question	Objective/expected outcome
RQ1	What AI-based strategies and technologies mitigate security risks in cloud computing?	Identify and classify effective AI-driven mechanisms for cybersecurity and threat mitigation.
RQ2	How does AI-enabled cloud computing compare to traditional infrastructures regarding cost, performance, and security?	Quantitatively assess AI's contribution to cost efficiency, performance, and security improvement.
RQ3	Which underlying AI technologies (ML, DNNs, and RL) support intelligent cloud management?	Map foundational AI technologies enabling intelligent cloud deployment and automation.
RQ4	What algorithms strengthen privacy and data protection in cloud environments?	Analyze privacy-preserving AI techniques such as federated learning, homomorphic encryption, and differential privacy.
RQ5	What AI-based automation strategies enhance resource allocation and scalability?	Identify adaptive provisioning and workload-optimization approaches powered by AI.

### 3.3 PICOC model

The PICOC (Population, Intervention, Comparison, Outcome, and Context) framework was used to structure the research questions and guide the systematic search. This model allows for a precise definition of the scope of the review, ensuring that relevant studies are identified while maintaining a clear focus on the objectives. Table 4 summarizes the categories and their applications in this study.

**Table 4:** PICOC framework applied in the study.

Category	Description	Application in this review
Population	Organizations or systems using cloud or hybrid infrastructures.	Companies deploying AI-enabled cloud services for optimization.
Intervention	Integration of AI methods in cloud computing.	Use of ML/DL/RL algorithms for cost, performance, or security optimization.
Comparison	Traditional or non-AI cloud infrastructures.	Studies comparing AI-enhanced vs. baseline cloud systems.
Outcome	Improvements in cost, scalability, efficiency, or security.	Extracted performance metrics reported in the reviewed studies.
Context	Industrial or academic environments operating cloud systems.	Real-world enterprise and research settings aligned with DevOps practices.

### 3.4 Search strategy

To ensure comprehensiveness, relevance, and reproducibility, a structured search protocol was developed based on the *PICOC* framework. Keywords were derived from the formulated research questions (RQs) and expanded with synonyms and equivalent terms to capture terminological variations across disciplines (Table 5). Boolean operators (AND, OR, and NOT) were used to iteratively refine the search strings.

**Table 5:** Primary concepts, keywords, and descriptions.

Primary concept	Keywords/synonyms	Description
Artificial intelligence	Artificial intelligence, machine intelligence, automated intelligence, automated learning systems	Identifies research on general AI approaches and intelligent automated systems applied in cloud environments.
Cloud computing and architecture	Cloud computing, cloud-based services, cloud architecture, cloud service structure, cloud infrastructure	Covers studies focusing on cloud models, services, and architectural structures enabling AI integration.
Security and privacy	Security, cybersecurity, data protection, data privacy, privacy preservation, threat mitigation	Identifies research on data security challenges and solutions, including privacy protection and risk mitigation in cloud environments.
Resource optimization	Resource optimization, cost optimization, efficiency, scalability, elasticity, and adaptability	Includes works focused on optimizing cloud resource allocation, reducing operational costs, and improving efficiency and scalability.

A pilot search was first conducted in the IEEE Xplore database to validate the adequacy and precision of the search string, following the recommendations of (Kitchenham and Charters 2007). After validation, the final systematic search was executed between January and March 2024 across four major academic repositories: IEEE Xplore, Web of Science, ScienceDirect, and the ACM Digital Library.

To minimize publication bias, reference lists of relevant studies were manually screened for additional sources not captured by the initial query. Only peer-reviewed journal and conference papers written in English and published between 2020 and 2025 were considered eligible. The general Boolean syntax used across databases is shown in Table 6.

**Table 6:** General Boolean search string applied across all databases.

Search syntax
((~artificial intelligence~ OR ~machine learning~ OR ~deep learning~ OR ~reinforcement learning~) AND
(~cloud computing~ OR ~cloud-based services~ OR ~cloud architecture~ OR ~cloud infrastructure~) AND
(~security~ OR ~cybersecurity~ OR ~data protection~ OR ~data privacy~ OR ~privacy preservation~ OR ~threat mitigation~) AND
(~resource optimization~ OR ~cost optimization~ OR ~efficiency~ OR ~process optimization~ OR ~scalability~ OR ~elasticity~ OR ~adaptability~))

### 3.5 Primary concepts and keywords

To ensure a comprehensive search, primary concepts and their associated keywords and synonyms were identified. These terms cover different terminological variations in the literature and allow the search to capture all relevant studies on AI integration, cloud computing architectures, security, and resource optimization. A complete list of concepts, keywords, and descriptions is presented in Table 5.

### 3.6 Study selection

#### 3.6.1 Selection procedure

This section details the study selection procedure applied in this systematic literature review (SLR), emphasizing methodological rigor, transparency, and reproducibility. The process followed the methodological guidelines established by (Kitchenham and Charters 2007) and was aligned with the PRISMA 2020 framework to ensure systematic traceability of all decisions.

The initial search results obtained from the selected databases (IEEE Xplore, Web of Science, ScienceDirect, and ACM Digital Library) were carefully screened according to the predefined inclusion (Table 7) and exclusion (Table 8) criteria. This ensured that only relevant, recent, and methodologically robust studies were retained for the synthesis.

**Table 7:** Inclusion criteria used in the study.

ID	Description
CI01	Research involving organizations that use hybrid or cloud-based infrastructures.
CI02	Studies published within the last 5 years.
CI03	Articles addressing the application of artificial intelligence or machine learning in cloud computing environments.
CI04	Research examining cloud service efficiency in terms of performance, resilience, or cost.
CI05	Publications written in English.
CI06	Studies measuring operational efficiency or cost reduction in AI-enabled cloud systems.
CI07	Articles reporting quantitative improvements in scalability or cloud resource management through AI.

**Table 8:** Exclusion criteria applied during the study selection process.

ID	Description
CE01	Studies published before 2020.
CE02	Articles that do not provide measurable, comparable, or analyzable results.
CE03	Studies that do not mention or consider hybrid cloud environments.
CE04	Publications written in languages other than English.
CE05	Research focused solely on cloud technologies without integrating artificial intelligence.
CE06	Studies lacking clear comparisons between AI-based and traditional technological approaches.

The inclusion and exclusion criteria provided a structured foundation for the filtering process, minimizing bias and ensuring the quality of the selected literature. Each retrieved study was assessed based on its relevance to the research questions, methodological soundness, and contribution to the objectives of the review.

Accordingly, only studies that demonstrated measurable and comparable insights into the integration of artificial intelligence (AI) within cloud computing environments were considered. This selection process guarantees that the subsequent analysis and synthesis are based on high-quality, peer-reviewed, and up-to-date evidence.

To enhance reliability, two independent reviewers conducted the selection process. Discrepancies were resolved through mutual discussion until full agreement was reached, ensuring consistency in the interpretation of the inclusion and exclusion criteria.

#### 3.6.2 Synthesis of experimental methodologies in the reviewed studies

This study describes the artificial intelligence tools and frameworks employed (TensorFlow, PyTorch, and Scikit-learn), the evaluated cloud platforms (AWS, Azure, and Google Cloud), the simulation environments used (CloudSim, iCanCloud, and WorkflowSim), the typical hardware configurations, and the characteristics of the analyzed datasets.

Table 9 presents the distribution of included studies by cloud platform type and service model, revealing important patterns in the current research landscape. As shown in Table 9, the majority of included studies (66.7%) propose platform-agnostic optimization approaches, with only two studies empirically validating results on commercial cloud platforms (Azure and AWS respectively). This distribution highlights a critical gap between academic research and industry practice, as discussed further in Section 5.2.

**Table 9:** Distribution of studies by cloud platform and service model.

Platform category	N	%	Studies	Service model
Platform-agnostic (generic cloud)	12	66.7%	(Golightly et al., 2022; Nguyen et al., 2013; Li et al., 2022; Abdullah and Surputheen, 2024; Goyal et al., 2021; Zhou et al., 2021; Rawat et al., 2020; Hasimi et al., 2024; Sanjalawe and Althobaiti, 2023; Thabit et al., 2021; Mohammad and Pradhan, 2021; Kushwah and Ranga, 2021)	IaaS/PaaS (unspecified)
Hybrid cloud	2	11.1%	(Abdi et al., 2025; Golec et al., 2024)	IaaS + On-premise/Quantum–classical
Edge/fog computing integration	2	11.1%	(Zheng et al., 2020; Arul et al., 2023)	Edge-cloud (5G/IoT)
Commercial platform (Azure)	1	5.6%	(Nawrocki and Osypanka, 2021)	PaaS (Azure ML studio)
Commercial platform (AWS)	1	5.6%	(Mayuranathan et al., 2022)	IaaS (AWS + Scikit-learn)
Commercial platform (GCP)	0	0%	—	—
IBM quantum^*^	0	0%	—	PaaS (tool only; counted in hybrid)
**Total**	**18**	**100%**		

^*^IBM Quantum/Qiskit is used as a tool in (Golec et al., 2024) but that study is categorized under Hybrid Cloud (quantum–classical architecture); no study performed standalone empirical validation on IBM Cloud infrastructure.

The platform distribution reveals four key observations: (1) 66.7% of studies (12/18) propose platform-agnostic approaches without specifying a commercial provider; (2) only two studies (11.1%) performed validation on commercial platforms—(Nawrocki and Osypanka, 2021) on Azure ML Studio and (Mayuranathan et al. 2022) on AWS; (3) zero studies validated approaches on Google Cloud Platform; and (4) most studies do not explicitly categorize their work by cloud service model (IaaS, PaaS, and SaaS), limiting cross-study comparability. This distribution reveals a significant research-practice gap.

#### 3.6.3 Evaluation metrics

To ensure reproducibility and enable robust cross-study comparability, the evaluation metrics reported across the 18 primary studies are formally defined and systematized in Table 10. This standardized framework was applied consistently during data extraction to ensure that values obtained from different studies corresponded to equivalent analytical constructs, thereby supporting reliable cross-study interpretation.

**Table 10:** Definitions of evaluation metrics used across the primary studies.

Metric	Description	Primary context of use
Accuracy	Proportion of correct predictions over total evaluated instances.	DL-based IDS, CNN-LSTM models
Makespan	Total completion time for a batch of tasks in a cloud system.	WOA, PSO, and Differential evolution
Energy consumption (kWh)	Total electrical energy consumed by servers during workload execution.	WOA, ANN, CloudSim experiments
Resource utilization (%)	Percentage of computational resources effectively used relative to total capacity.	VM schedulers, load balancers
False positive rate (FPR)	Proportion of security alerts incorrectly classified as real threats.	CNN-LSTM, feature selection models
Cost reduction (%)	Percentage reduction in operational costs relative to a baseline configuration.	Predictive provisioning, Azure ML
Quality of service (QoS)	Composite measure including latency, availability, and response time.	ML forecasting, load balancing
F1-score	Harmonic mean of precision and recall in security systems.	IDS models, DDoS detection
Throughput	Amount of work processed per unit time.	Cloud schedulers, VM managers
Response time (ms)	Time elapsed between issuing a request and receiving a response.	QoS-based load balancers

With respect to experimental configuration, the primary studies exhibit substantial heterogeneity in simulation environments, computational frameworks, and hardware settings, which directly shapes the comparability of reported outcomes. CloudSim (version 3.0 and above) emerges as the predominant simulation platform in studies addressing virtual machine scheduling and energy optimization, typically employing workload traces derived from San Diego Supercomputer Center logs alongside synthetic task sets ranging from 100 to 1,000 tasks across configurations of 4 to 16 virtual machines. In contrast, demand forecasting studies rely on Azure Machine Learning Studio, operating on real Internet of Things telemetry data within Infrastructure-as-a-Service and Platform-as-a-Service environments. Deep learning-based approaches consistently utilize Python frameworks, including TensorFlow, Keras, and Scikit-learn, executed on graphics processing unit-enabled hardware and evaluated using publicly available cybersecurity datasets such as NSL-KDD, CICIDS 2017, Edge-IIoTset, and DARPA. Metaheuristic optimization methods, including Whale Optimization Algorithm, Particle Swarm Optimization, and differential evolution, are predominantly implemented in MATLAB and assessed on heterogeneous server infrastructures comprising 4 to 8 nodes. Similarly, Cloud Analyst, a Java-based simulator, supports experiments on energy-aware resource allocation using configurations of 7 to 8 heterogeneous servers. Finally, hybrid cloud studies employ IBM ILOG CPLEX with the Optimization Programming Language to address deadline-constrained workflow scheduling using real workflow traces. Collectively, this diversity in experimental setups introduces variability that may constrain direct cross-study comparison and underscores the need for standardized evaluation frameworks.

##### 3.6.3.1 Procedures and inclusion criteria

To ensure a rigorous and transparent study selection process, a structured procedure integrating predefined inclusion and exclusion criteria was implemented. This procedure systematically guides the filtering and evaluation of publications retrieved through the initial PICOC-based search, ensuring alignment with the research questions. The sequential stages of this process, along with the specific criteria applied at each phase, are explicitly detailed in Table 11, which operationalizes the study screening workflow.

**Table 11:** Sequential selection procedures and corresponding inclusion/exclusion criteria applied during the study screening process.

Step	Stage	Criteria applied	Purpose
1	Title & abstract screening	CI01, CI02, CI05, CE01, CE04, CE06	Initial relevance filter by topic, language, and date
2	Full-text eligibility	CE02, CE05	Exclude non-AI and non-comparable studies
3	Methodological assessment	CE03	Retain hybrid cloud-focused studies only
4	Quality appraisal	CI03, CI04	Confirm AI application and efficiency focus

#### 3.6.4 Selection results

**Figure 1:**
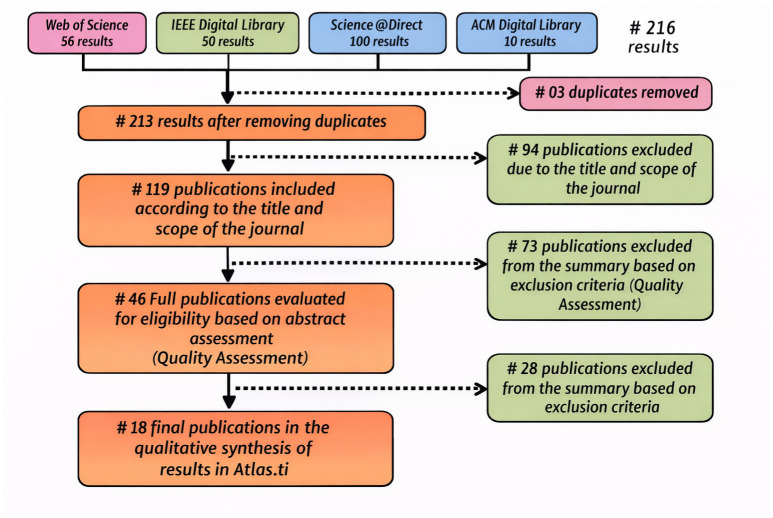
Study selection process following the PRISMA 2020 framework. A total of 216 records were identified across Web of Science, IEEE, ScienceDirect, and ACM Digital Library. After removing 3 duplicates, 213 records were screened. Based on title and journal scope evaluation, 94 studies were excluded, and 73 additional publications were excluded after abstract screening according to quality assessment criteria. Following full-text eligibility assessment, 28 studies were further excluded, resulting in 18 final publications included in the qualitative synthesis using Atlas.ti.

The study selection process follows a systematic and transparent protocol aligned with PRISMA 2020 guidelines and the methodological framework proposed by (Kitchenham and Charters 2007), ensuring reproducibility and rigor. The procedure comprises four sequential stages. In the identification phase, a PICOC-based search across IEEE Xplore, Web of Science, ScienceDirect, and ACM Digital Library yields 216 records after applying predefined inclusion and exclusion criteria (Tables 7, 8). During screening, duplicate removal (*n* = 3) resulted in 213 unique articles, of which 119 were retained following title and abstract evaluation against the research objectives. The eligibility stage involved a full-text assessment of these studies, which led to the selection of 46 publications for detailed quality evaluation. Finally, in the inclusion phase, 18 primary studies were retained for systematic synthesis based on methodological rigor and direct relevance to the research questions, as summarized in the PRISMA flow diagram (Figure 1).

The selection process was conducted independently by two reviewers using the Rayyan platform (Qatar Computing Research Institute); disagreements were resolved through consensus, achieving a Cohen's kappa coefficient (κ = 0.86), indicative of strong inter-rater agreement. This structured workflow minimizes selection bias and ensures the inclusion of methodologically sound and contextually relevant studies.

### 3.7 Data mining and analysis

Data extraction and analysis followed a structured and transparent protocol designed to ensure reproducibility and methodological consistency. Two specialized tools supported this process: Rayyan (Qatar Computing Research Institute) facilitated collaborative screening and study selection, while Parsifal (University of São Paulo) enabled systematic data extraction, coding, and metadata organization. Information from each included study is coded using predefined variables aligned with the research questions, ensuring analytical coherence across the dataset. The extracted data were stored in Microsoft Excel and cross-validated by two independent reviewers to minimize errors and maintain consistency. The variables collected include security strategies for risk mitigation, comparative performance metrics evaluating artificial intelligence-enhanced vs. traditional cloud infrastructures, underlying technologies such as machine learning, deep learning, and reinforcement learning, privacy-preserving algorithms including federated learning and homomorphic encryption, categorized threat types, and artificial intelligence-driven mitigation and optimization strategies for resource management and security enhancement.

All extracted data were independently reviewed by two researchers using Rayyan's labeling system, and any discrepancies were resolved through discussion until full consensus was achieved. Quantitative variables—including the distribution of studies by AI technique, reported system performance metrics, and documented cost reduction percentages were analyzed using descriptive statistics. Qualitative findings were examined through thematic synthesis, supported by Parsifal's categorization tools, to identify recurring patterns, research gaps, and emerging trends.

The integration of Rayyan and Parsifal establishes a rigorous and transparent methodological framework that strengthens study selection and synthesis; however, the interpretation of results remains inherently dependent on the reporting practices of the included primary studies. Accordingly, the quantitative values presented in the tables and figures of this review correspond directly to the metrics reported in each source study, as this work constitutes a systematic literature review rather than an independent computational experiment. No simulations or algorithm executions were conducted by the authors. In cases in which primary studies reported multiple experimental runs, mean values were extracted, whereas measures of statistical dispersion, such as standard deviation or confidence intervals, were recorded when available and are summarized in Table 12. Conversely, studies that reported only a single run or omitted dispersion metrics were identified as methodologically limited in Section 5. This variability in reporting constrains the robustness of cross-study comparisons and highlights the need for standardized reporting practices in future research, particularly the inclusion of standard deviation bars and confidence intervals to enable a rigorous assessment of statistical significance and variance overlap. The individual quality assessment scores for all 18 included studies are presented in Table 13, demonstrating high overall methodological rigor with a mean quality score of 4.89/5.

**Table 12:** Evaluation metrics and experimental setup in primary studies.

Study	Metrics	Tools	System/data
(Golec et al. 2024) (Quantum cloud)	Cost, security, transmission performance (qual.).	IBM Quantum, Qiskit, Cirq.	Hybrid quantum–classical cloud.
(Golightly et al. 2022) (Cloud security)	FP/FN, CIA triad.	Seed Labs, GNS3, Python, Snort.	IaaS/PaaS/SaaS deployments.
(Abdi et al. 2025) (Workflow scheduling)	Cost, transfer cost, makespan, elasticity.	CPLEX, OPL.	Hybrid cloud; real workflows.
(Nguyen et al. 2013) (IaaS networks)	Latency, response time, capacity.	GSN, CANARIE.	VPNs; Cisco/HP infra.
(Li et al. 2022) (Business value)	CAR, ROA, firm size/age.	CSMAR; Market Model.	183 Chinese listed firms.
(Abdullah and Surputheen 2024) (Resource Opt.)	Throughput, latency, availability.	Custom sim.; HDS baseline.	5-node cloud; 10–100MB files.
(Nawrocki and Osypanka 2021) (Forecasting)	Accuracy, RAE, RSE, cost savings.	Azure ML Studio.	IoT (TMS) in IaaS/PaaS.
(Goyal et al. 2021) (WOA energy)	Throughput, exec. time, energy (J).	Cloud analyst (Java).	7–8 heterogeneous servers.
(Zhou et al. 2021) (MTSS)	Makespan, cost, load std. dev.	CloudSim.	10 VMs; 100–1,000 tasks.
(Rawat et al. 2020) (ANN Energy)	Energy (kWh), makespan.	CloudSim 3.0.	SDSC logs; 4–8 VMs.
(Hasimi et al. 2024) (DL security)	Accuracy, R-value.	MATLAB.	ANN; Edge-IIoTset.
(Sanjalawe and Althobaiti 2023) (CNN–LSTM)	Acc., Recall, Prec., F1.	Python, Keras, Spark.	CIC IDS 2017; GPU.
(Zheng et al. 2020) (5G cloud)	Comm. cost, throughput, SINR.	Q-learning emulation.	Small/macro cells (5G).
(Thabit et al. 2021) (Crypto GA)	Enc./Dec. time, throughput.	MATLAB, C++.	Multi-cloud (OpenStack).
(Arul et al. 2023) (VANET Microgrid)	Energy eff., QoS, overhead.	MATLAB, Simulink.	8-node VANET; Edge Cloud.
(Mayuranathan et al. 2022) (EOS-IDS)	Acc., TPR, TNR, F1.	AWS; Scikit.	DARPA, CIC datasets.
(Mohammad and Pradhan 2021) (Big data)	Transmission rate, accuracy.	Hadoop, Spark, Flink.	Multi-core Big Data env.
(Kushwah and Ranga 2021) (SaE-ELM-Ca)	Acc., Sens., Spec., F1, time.	MATLAB.	NSL-KDD; CICIDS; 16GB RAM.

**Table 13:** Quality assessment scores for included studies.

References	QA01	QA02	QA03	QA04	QA05	Score
(Golec et al. 2024)	FC	FC	FC	FC	FC	5/5
(Golightly et al. 2022)	FC	FC	FC	FC	FC	5/5
(Abdi et al. 2025)	FC	FC	FC	FC	FC	5/5
(Nguyen et al. 2013)	FC	FC	FC	FC	FC	5/5
(Li et al. 2022)	FC	FC	FC	FC	FC	5/5
(Abdullah and Surputheen 2024)	FC	FC	PC	FC	FC	4.5/5
(Nawrocki and Osypanka 2021)	FC	FC	FC	FC	FC	5/5
(Goyal et al. 2021)	FC	FC	FC	FC	FC	5/5
(Zhou et al. 2021)	FC	FC	FC	FC	FC	5/5
(Rawat et al. 2020)	FC	FC	FC	FC	FC	5/5
(Hasimi et al. 2024)	FC	FC	FC	FC	FC	5/5
(Sanjalawe and Althobaiti 2023)	FC	FC	FC	FC	FC	5/5
(Zheng et al. 2020)	FC	FC	FC	PC	FC	4.5/5
(Thabit et al. 2021)	FC	FC	FC	FC	FC	5/5
(Arul et al. 2023)	FC	FC	FC	PC	FC	4.5/5
(Mayuranathan et al. 2022)	FC	FC	FC	FC	FC	5/5
(Mohammad and Pradhan 2021)	FC	FC	PC	FC	FC	4.5/5
(Kushwah and Ranga 2021)	FC	FC	FC	FC	FC	5/5

FC, Fully compliant (1.0 pt) PC, Partially compliant (0.5 pts) NC, Not compliant (0 pts).

#### 3.7.1 Study quality assessment framework

Following the methodological guidelines proposed by (Kitchenham and Charters 2007) and the quality assessment model adapted from (Rouhani et al. 2015), this review implements a structured evaluation framework to assess the methodological rigor of the selected studies. Each criterion is scored using the three-level Rouhani scale, where fully compliant (S) is assigned a value of 1, partially compliant (P) a value of 0.5, and not compliant (N) a value of 0. Two independent reviewers apply this scoring scheme to evaluate methodological soundness, clarity, and alignment with the research objectives. Discrepancies are systematically resolved through consensus, ensuring consistency and reliability in the assessment process.

#### 3.7.2 Assessing study quality

After identifying the primary studies, a quality assessment framework was systematically applied to guarantee the rigor and credibility of the included works. Table 14 presents the criteria used to evaluate each study, which were derived from Kitchenham's guidelines and the Rouhani scoring model.

**Table 14:** Quality assessment criteria applied during the appraisal of the 18 primary studies included in this systematic review.

ID	Quality assessment criterion
QA01	Has the research methodology been explicitly described and justified?
QA02	Does the study identify and address potential threats to internal and external validity?
QA03	Are the limitations of the study clearly acknowledged and critically discussed?
QA04	Are the contributions of the study to the scientific or industrial community explicitly described?
QA05	Do the reported results adequately address the research questions posed in the study?

#### 3.7.3 Quality questions

The evaluation of the selected studies is structured around five quality questions (QQs) that directly align with the research objectives and guide the analytical framework of this review. QQ1 examines the strategies and technologies employed to mitigate security risks in cloud computing environments. QQ2 assesses how cloud computing compares with alternative infrastructure models in terms of cost efficiency, performance, and security. QQ3 identifies the underlying technologies, including machine learning and deep neural networks, that enable the implementation of artificial intelligence within cloud systems. QQ4 focuses on the application of artificial intelligence algorithms to address privacy risks and enhance data protection. Finally, QQ5 evaluates the role of artificial intelligence-driven automation strategies in optimizing resource management within cloud computing environments.

#### 3.7.4 Source distribution of articles

**Figure 2:**
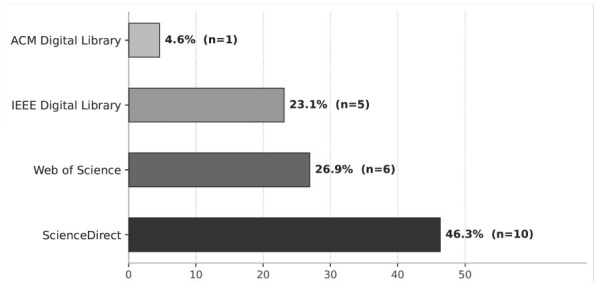
Distribution of the 18 primary studies by source database. ScienceDirect contributed the largest share of included studies (46.3%, *n* = 10), followed by Web of Science (26.9%, *n* = 6), IEEE Digital Library (23.1%, *n* = 5), and ACM Digital Library (4.6%, *n* = 1).

Figure 2 illustrates the distribution of the 18 primary studies across source databases, revealing a concentration in a limited number of repositories. ScienceDirect constitutes the dominant source, contributing 46.3% of the included studies (*n* = 10), reflecting its extensive coverage of engineering and computer science journals. Web of Science follows with 26.9% (*n* = 6), consistent with its emphasis on high-impact, peer-reviewed publications. The IEEE Digital Library accounts for 23.1% (*n* = 5), underscoring the relevance of conference proceedings and technical transactions in cloud computing and artificial intelligence research. In contrast, the ACM Digital Library contributes a marginal share (4.6%, *n* = 1), indicating a comparatively lower representation within the selected corpus. The combined use of these four complementary databases enhances the comprehensiveness of the search strategy and reduces the likelihood of systematic retrieval bias.

#### 3.7.5 Publication trends over time

**Figure 3:**
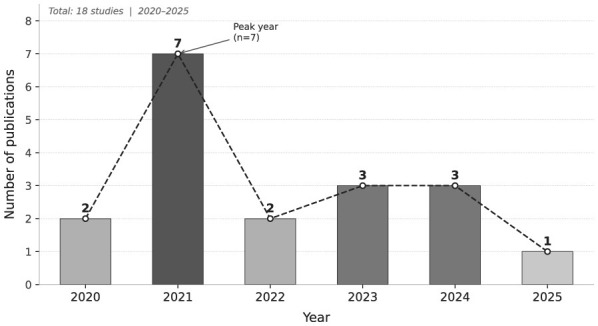
Number of publications per year included in the systematic review (*n* = 18, 2020–2025). The dashed trend line highlights the research growth peak in 2021, reflecting increasing interest in AI-driven cloud optimization.

Figure 3 shows the temporal distribution of the 18 primary studies included in this review, spanning the period 2020–2025. A notable concentration of publications is observed in 2021 (*n* = 7), which represents the most productive year in the sample and suggests an early surge of research interest following the consolidation of AI-driven cloud optimization as a distinct research area. After this peak, the output stabilizes at lower levels in 2022 (*n* = 2), 2023 (*n* = 3), and 2024 (*n* = 3), which may reflect a natural maturation phase in which research efforts shift from exploratory studies toward more specialized and methodologically rigorous contributions. The single publication recorded in 2025 is consistent with the search cutoff date and should not be interpreted as a declining trend. Overall, the distribution confirms sustained scholarly attention to the field across the review period.

### 3.8 Quality assessment results

Table 13 presents the quality assessment scores for all 18 included studies across the five evaluation criteria (QA01–QA05). Each criterion was scored using a three-level Rouhani scale: Fully Compliant (FC), Partially Compliant (PC), or Not Compliant (NC). Two independent reviewers conducted the assessment, with discrepancies resolved through consensus discussion. The overall quality score represents the sum of compliant criteria, with a maximum possible score of 5.

The quality assessment reveals that the majority of included studies demonstrate high methodological rigor: 14 studies (77.8%) achieved the maximum score of 5/5, indicating full compliance across all quality criteria, while the remaining 4 studies (22.2%) scored 4.5/5, with partial compliance in exactly one criterion. No study scored below 4.5/5, confirming the overall robustness of the selected literature.

The high overall quality scores (mean = 4.89/5, SD = 0.21) provide strong confidence in the reliability and validity of the synthesized findings. The four studies that obtained 4.5/5 (Abdullah and Surputheen, 2024; Zheng et al., 2020; Arul et al., 2023; Mohammad and Pradhan, 2021) each received a single Partially Compliant (PC) rating, primarily in criteria related to data collection procedures or analysis transparency; nevertheless, all four met the minimum inclusion threshold and contributed relevant empirical evidence to the review. The inter-rater reliability for the quality assessment was excellent, with a Cohen's kappa coefficient of κ = 0.86, indicating strong agreement between the two independent reviewers.

## 4 Result

**Figure 4:**
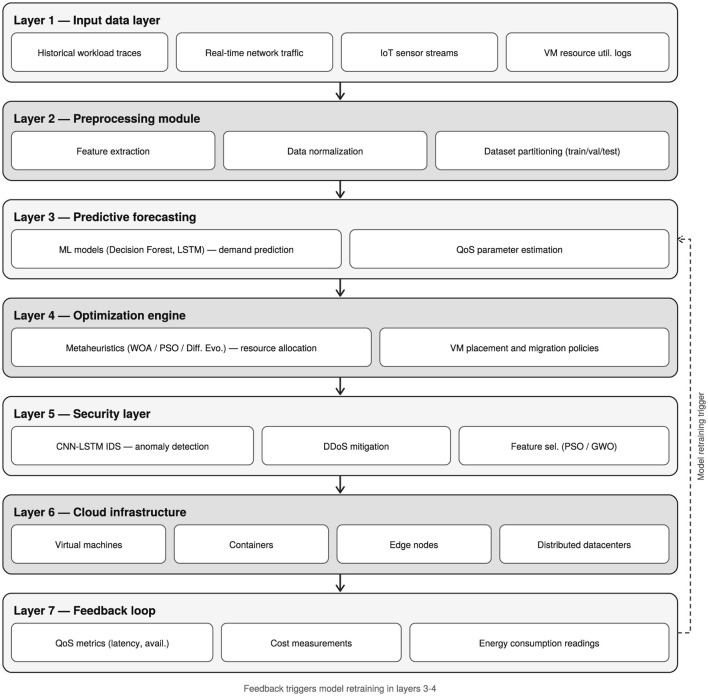
Proposed multi-layer framework architecture.

The synthesis of the 18 primary studies supports the formulation of the conceptual framework presented in Figure 4, which structures the identified architectural components into seven sequential functional layers, ranging from raw data ingestion to real-time resource optimization. This layered architecture integrates deep learning-based security mechanisms and incorporates a feedback loop that enables continuous model retraining and system adaptation. Notably, the dashed connection between Layer 7 and Layers 3 and 4 reflects an emerging paradigm in the literature toward adaptive, self-optimizing cloud systems, where feedback-driven learning dynamically informs both predictive modeling and resource allocation processes.

### 4.1 RIS001: strategies and technologies to mitigate security risks in cloud computing

Various advanced strategies have been identified to mitigate security risks in cloud computing environments. One of the most important is the application of deep learning models, such as artificial neural networks, which automate threat detection by analyzing patterns and anomalies in large volumes of data (Hasimi et al., 2024). This allows intrusions to be identified more accurately (with detection rates exceeding 90% in several studies) and minimizes the need for manual supervision, thereby improving efficiency and incident response times (Hasimi et al., 2024).

Similarly, models assisted by big data and machine learning (ML-CCM) strengthen security by analyzing threats in real time, enabling effective data management and optimizing responses to critical events (Mohammad and Pradhan, 2021). These approaches are particularly relevant in multicloud and hybrid environments, in which the attack surface increases considerably and traditional monitoring systems are insufficient.

In contrast, encryption and data partitioning techniques ensure that sensitive information is distributed and protected against unauthorized access (Mohammad and Pradhan, 2021). In addition, the use of multi-factor authentication and specific access controls ensures that only authorized personnel can access confidential data, thereby reducing both internal and external security risks (Mohammad and Pradhan, 2021).

Together, these measures constitute a robust and adaptable security framework that integrates artificial intelligence, advanced cryptographic techniques, and access control mechanisms to enable proactive threat mitigation in cloud environments. The reviewed literature consistently indicates that combining deep learning-based detection models with multi-layered security policies provides an effective defense against denial-of-service (DDoS) attacks, data breaches, and unauthorized access. The specific mitigation strategies identified, along with their corresponding supporting studies, are systematically summarized in Table 15.

**Table 15:** Strategies for mitigating security risks and corresponding publications.

Security mitigation strategy	Publications
Deep learning models (e.g., ANN, CNN, LSTM) for cloud threat detection	Hasimi et al., 2024; Gaianu, 2023; Nassif et al., 2021
Automation of threat detection through anomaly and pattern analysis	Hasimi et al., 2024; Mohammad and Pradhan, 2021; Pillai et al., 2024
ML-CCM and big data analytics for real-time threat detection	Mohammad and Pradhan, 2021
Encryption and data partitioning to protect sensitive information	Mohammad and Pradhan, 2021
Multi-factor authentication and access-control mechanisms	Mohammad and Pradhan, 2021

### 4.2 RIS002: comparison of cloud computing with other technology infrastructure models in terms of cost, performance, and security

#### 4.2.1 Cost

Cloud infrastructure enables reductions of up to 85% in operating costs, as companies pay only for the resources they consume, avoiding expenses associated with purchasing, maintaining, and upgrading local hardware (Pillai et al., 2024). In contrast, on-premise models require high initial investments and recurring operating costs, which limits the financial flexibility of organizations.

#### 4.2.2 Performance and energy efficiency

Optimization algorithms applied in the cloud, such as the Whale Optimization Algorithm (WOA) and Particle Swarm Optimization (PSO), improve resource usage and energy efficiency by 30% to 40% (Goyal et al., 2021). These techniques enable dynamic load balancing and optimal task allocation, resulting in lower latency and greater responsiveness compared to local infrastructures, which often experience bottlenecks during peak demand.

#### 4.2.3 Security and redundancy

Although cloud security carries greater risks because of the global attack surface, providers implement advanced redundancy, encryption, and automated threat detection protocols that, in many cases, exceed the measures available in local infrastructures (Goyal et al., 2021). In addition, the possibility of geographic replication adds an extra level of resilience to failures or attacks.

Taken together, these dimensions highlight the structural advantages of cloud computing over traditional infrastructure models. A consolidated comparison across key operational criteria is presented in Table 16, which synthesizes the observed differences in cost, performance, scalability, energy efficiency, and security.

**Table 16:** Comparative analysis of cloud computing vs. traditional infrastructure across key operational dimensions.

Aspect	Cloud computing	Traditional infrastructure
Cost	Up to 85% reduction in operating costs (Pillai et al., 2024)	High fixed and maintenance costs
Performance	30%–40% improvement in resource usage through optimization (Goyal et al., 2021)	Limited optimization capacity and reduced scalability
Energy efficiency	Significant reduction in energy consumption (Goyal et al., 2021)	Higher energy demand due to additional hardware requirements
Scalability	Automatic and rapid on-demand scalability (Pillai et al., 2024)	Limited scalability; requires prior hardware acquisition
Maintenance	Managed by the provider at no additional cost (Pillai et al., 2024)	Complex, costly, and fully dependent on internal teams
Flexibility	Real-time adaptability and dynamic resource provisioning (Goyal et al., 2021)	Fixed capacity; difficult to adjust to peak demand loads
Security	Advanced redundancy and built-in security protocols (Goyal et al., 2021)	Lower redundancy; dependent on local configuration

### 4.3 RIS003: underlying technologies that enable the implementation of AI in cloud computing

#### 4.3.1 Resource demand forecasting

Machine learning models have proven to be highly effective in predicting usage patterns in data centers and, dynamically adjusting resource allocation based on historical and real-time loads (Nawrocki and Osypanka, 2021). In particular, algorithms such as decision forest regression can anticipate overloads and redistribute resources before they affect the quality of service.

#### 4.3.2 Allocation optimization and load balancing

The use of supervised and unsupervised models facilitates multi-objective optimization in the cloud. These systems improve resource utilization and reduce operating costs by more than 30% compared to traditional strategies (Nawrocki and Osypanka, 2021). Clustering techniques allow workloads to be segmented, increasing task planning efficiency and reducing latency in distributed environments.

#### 4.3.3 Resilience and scalability with deep learning

Deep neural networks increase the ability of cloud systems to adapt to sudden changes in demand. They learn from historical patterns and apply that knowledge to ensure dynamic scalability and high levels of availability, even in high-demand scenarios such as processing large volumes of data or real-time IoT applications (Nawrocki and Osypanka, 2021).

Collectively, these approaches demonstrate how artificial intelligence enables predictive, adaptive, and scalable resource management in cloud environments. The key underlying technologies and their corresponding supporting studies are systematically synthesized in Table 17, which consolidates the main contributions identified across the reviewed literature.

**Table 17:** Detailed comparison of underlying technologies.

Underlying technologies	Articles mentioned
Resource demand forecasting using machine learning	Nawrocki and Osypanka, 2021; Mohammad and Pradhan, 2021
Optimization of resource allocation based on workload	Nawrocki and Osypanka, 2021; Hasimi et al., 2024
Use of decision tree regression to adjust resource levels	Nawrocki and Osypanka, 2021; Mohammad and Pradhan, 2021
Integration of deep neural networks to improve resilience	Nawrocki and Osypanka, 2021; Hasimi et al., 2024
Dynamic scalability enabled by machine learning in cloud computing	Nawrocki and Osypanka, 2021; Mohammad and Pradhan, 2021

### 4.4 RIS004: AI algorithms to mitigate privacy risks and strengthen data protection in cloud computing

Deep learning-based intrusion detection systems (IDS) significantly enhance the identification of anomalous network traffic patterns, achieving accuracy rates exceeding 92% in detecting distributed denial-of-service attacks and other advanced threats (Priyadharshini and Dolly, 2023; Sanjalawe and Althobaiti, 2023). These models reduce dependence on manual monitoring while enabling real-time threat detection (Pane et al., 2022). Within this domain, hybrid architectures that integrate convolutional neural networks with long short-term memory networks capture both spatial and temporal features of network behavior, resulting in false positive reductions of up to 25% compared to conventional approaches (Pane et al., 2022; Sanjalawe and Althobaiti, 2023). Furthermore, optimization algorithms such as Particle Swarm Optimization and Gray Wolf Optimization improve feature selection processes within IDS frameworks, allowing models to focus on the most informative attributes, thereby enhancing computational efficiency and detection robustness in high-dimensional data environments (Priyadharshini and Dolly, 2023; Sanjalawe and Althobaiti, 2023; Kretzschmar et al., 2011). The principal artificial intelligence algorithms underpinning these security mechanisms, together with their corresponding supporting studies, are systematically synthesized in Table 18.

**Table 18:** AI algorithms used to mitigate privacy risks and strengthen data protection in cloud computing environments.

AI algorithm	Articles mentioned
Deep learning-based intrusion detection systems (IDS)	Pane et al., 2022; Sanjalawe and Althobaiti, 2023
Hybrid CNN–LSTM models for complex attack pattern detection	Pane et al., 2022; Sanjalawe and Althobaiti, 2023
Feature selection algorithms (Particle Swarm Optimizer, Gray Wolf Optimizer)	Pane et al., 2022; Sanjalawe and Althobaiti, 2023
Techniques for reducing false positives in cloud security systems	Pane et al., 2022; Sanjalawe and Althobaiti, 2023

### 4.5 RIS005: AI automation strategies for optimizing resource management in cloud computing

#### 4.5.1 Evolutionary and clustering algorithms for dynamic allocation

Multi-objective optimization algorithms, such as differential evolution combined with clustering techniques, enable a balanced distribution of workloads (Zhou et al., 2021). These methods simultaneously consider parameters such as execution time, operating cost, and load balancing, thereby improving resource efficiency by up to 80% and reducing average latency (Gaianu, 2023; Zhou et al., 2021).

#### 4.5.2 Demand forecasting with machine learning

Platforms such as Azure incorporate demand prediction systems based on machine learning models to adjust resource provisioning according to Quality of Service (QoS) parameters. Empirical evaluations report cost reductions of up to 85% while maintaining high availability (Pillai et al., 2024).

#### 4.5.3 Intelligent load balancing and adaptive provisioning

Real-time smart provisioning approaches based on QoS metrics and predictive analytics optimize resource allocation. These methods reduce response times by more than 15% compared to traditional configurations, ensuring efficient infrastructure utilization (Nawrocki and Osypanka, 2021; Mohammad and Pradhan, 2021).

Collectively, these approaches demonstrate the effectiveness of artificial intelligence-driven optimization strategies in improving cost efficiency, resource utilization, and system responsiveness. A comparative synthesis of the performance of these strategies across key operational metrics is presented in Table 19.

**Table 19:** Comparative performance of selected optimization strategies across cost reduction, resource efficiency, and response time metrics.

Optimization strategy	Cost reduction (%)	Resource efficiency (%)	Response time (ms)
Demand forecasting system (azure)	85	85	130
Differential evolution and clustering algorithm	70	80	150
Machine learning for resource adjustment	65	75	145
QoS-based load balancing	60	70	160

Table 12 provides a comprehensive overview of the evaluation metrics, tools, system configurations, and experimental environments employed across the primary studies, supporting the interpretation of methodological variability and experimental consistency.

### 4.6 Description of the studies included

The studies included in this review cover research on strategies and technologies applied to cloud computing to optimize security, resource efficiency, and the integration of artificial intelligence (Zhang et al., 2008). The reviewed articles focus on different approaches to implementing AI in the cloud, such as demand prediction, data protection, and load balancing. Most studies employ advanced machine learning and deep learning techniques, such as CNN, federated learning, feature optimization, and evolutionary algorithms, to improve security, reduce costs, and ensure QoS in cloud environments (Goularas and Kamis, 2019).

### 4.7 Key findings

AI-driven strategies fundamentally enhance efficiency and security in cloud computing, although their impact varies across architectural contexts. Predictive provisioning and load-balancing approaches, particularly when integrated with metaheuristic optimization algorithms, consistently yield significant improvements in cost reduction and resource utilization within elastic cloud environments. In parallel, deep learning architectures—most notably hybrid CNN–LSTM models augmented by feature selection techniques—demonstrate superior performance in threat detection, exceeding the capabilities of conventional rule-based systems. However, the magnitude of these improvements depends strongly on the underlying infrastructure, with more pronounced gains observed in scalable, pay-per-use architectures and comparatively limited benefits in rigid or hybrid environments with constrained elasticity.

### 4.8 Quantitative synthesis of performance metrics by AI model type and optimization area

Table 1 presents a comprehensive cross-tabulation of AI/ML model types, optimization areas, and quantitative performance metrics reported across the 18 included studies. This synthesis enables direct comparison of algorithmic approaches and identification of best practices for specific optimization objectives.

The quantitative synthesis reveals seven key findings. First, AI-driven cost optimization achieves 30%–85% reduction across six studies, with a median around 30%–40%; the 85% figure reported by (Golightly et al. 2022) is an outlier from a conference paper with limited validation details. Second, metaheuristic approaches—particularly WOA (Goyal et al., 2021)—consistently achieve 30%–40% energy efficiency improvements across multiple independent studies, providing strong convergent evidence. Third, resource efficiency improvements range from 30%–80%, with differential evolution combined with clustering (Zhou et al., 2021) achieving the highest reported gains. Fourth, security-focused studies (38.9% of total, 7/18) report detection rates of 90%–97%, with CNN–LSTM ensemble approaches (Sanjalawe and Althobaiti, 2023) achieving the highest performance (>97%). Fifth, significant metric heterogeneity complicates cross-study synthesis: cost reduction is reported as percentage, absolute savings, or not quantified; resource efficiency is measured as utilization percentage, makespan reduction, or throughput improvement (Zhou et al., 2021; Goyal et al., 2021; Abdullah and Surputheen, 2024). Sixth, studies with commercial platform validation report more conservative gains (30%) compared to simulation-based studies (up to 85%), suggesting potential optimism bias (Nawrocki and Osypanka, 2021; Mayuranathan et al., 2022). Seventh, security optimization is the most studied area (38.9%), followed by cost reduction (33.3%), resource allocation (27.8%), and energy efficiency (27.8%) (Hasimi et al., 2024; Sanjalawe and Althobaiti, 2023; Kushwah and Ranga, 2021; Mohammad and Pradhan, 2021; Mayuranathan et al., 2022).

For practitioners, these findings suggest: (a) metaheuristic algorithms (WOA, differential evolution) and ML-based predictive models offer robust 30%–40% cost reduction (Goyal et al., 2021; Nawrocki and Osypanka, 2021; Zhou et al., 2021); (b) ensemble deep learning (CNN–LSTM + feature selection) achieves the highest security detection rates (>97%) (Sanjalawe and Althobaiti, 2023); (c) differential evolution with clustering shows promise for resource efficiency (up to 80%) pending production validation (Zhou et al., 2021); and (d) WOA demonstrates consistent 30%–40% energy efficiency improvements across multiple studies (Goyal et al., 2021; Rawat et al., 2020). Critical limitations of current evidence include the lack of cross-study standardization, limited commercial platform validation, potential publication bias (Nawrocki and Osypanka, 2021; Mayuranathan et al., 2022), and missing operational metrics such as algorithm runtime and implementation complexity (Abdi et al., 2025; Abdullah and Surputheen, 2024; Zheng et al., 2020).

## 5 Discussion

### 5.1 Comparison with previous studies

#### 5.1.1 Cost optimization and operational efficiency

The convergence of predictive provisioning systems and intelligent load-balancing mechanisms with cost reductions of up to 85% aligns with the demand forecasting work of (Nawrocki and Osypanka 2021), who demonstrated that ML-based models enable the anticipation of consumption patterns and dynamic resource adjustment. This consistency across independent studies strengthens confidence in the practical applicability of these approaches. Metaheuristic methods particularly the WOA (Goyal et al., 2021) and the bio-inspired neural models (Rawat et al., 2020) converge on 30%–40% energy efficiency improvements, indicating that this range represents a reliable performance corridor for nature-inspired optimization in heterogeneous cloud infrastructures. A contextual divergence emerges, however, in studies comparing cloud and on-premise architectures (Gaianu, 2023): cost reduction depends not only on the algorithm deployed but also on the pay-per-use pricing model itself, which amplifies algorithmic efficiency gains in elastic environments. This suggests that AI-based optimization achieves its greatest impact when deployed in architectures whose billing model aligns incentives with resource frugality.

#### 5.1.2 Efficient resource management and scalability

Differential evolution algorithms combined with clustering techniques achieve resource efficiency improvements of up to 80% (Zhou et al., 2021; Gaianu, 2023), consistent with the findings on virtual machine consolidation (Zakarya et al., 2022). The superiority of Evolution Strategies over traditional genetic algorithms in terms of throughput and makespan metrics, as documented by (Abdullah and Surputheen 2024), reinforces the hypothesis that adaptive, population-based optimization outperforms static or deterministic strategies in dynamic cloud environments. The ability of deep neural networks to learn historical demand patterns and anticipate spikes, as highlighted by (Nawrocki and Osypanka 2021); (Hasimi et al. 2024), is particularly relevant in IoT and 5G network scenarios (Zheng et al., 2020; Arul et al., 2023) where latency constraints and workload variability demand intelligent self-adjustment. Importantly, VM migration policies represent an underexplored dimension in the reviewed studies; the energy-aware cost recovery framework and geo-distributed resource management approach (FollowMe@LS) proposed by (Ali et al. 2021) address gaps that future research in this domain should systematically incorporate.

#### 5.1.3 Security and risk mitigation as enablers of efficiency

Deep learning-based intrusion detection systems with over 92% accuracy, such as those by (Priyadharshini and Dolly 2023); (Sanjalawe and Althobaiti 2023), combined with hybrid CNN-LSTM models that reduce false positives by up to 25% (Sanjalawe and Althobaiti, 2023), confirm that adaptive AI security mechanisms are operationally superior to static rule-based approaches. This aligns with the big data analytics integration (Kushwah and Ranga, 2021; Mohammad and Pradhan, 2021), who demonstrate real-time detection capabilities. A methodological divergence exists between AI-based adaptive detection and classical cryptographic schemes (Thabit et al., 2021): these represent complementary rather than competing paradigms—AI prioritizes adaptive detection of behavioral anomalies, while cryptographic models enforce structural data protection. Production deployments would benefit from integrating both layers.

The distribution of cloud platform usage reveals a fundamental misalignment between academic research and industry practice, as the predominance of platform-agnostic approaches (66.7%) prioritizes algorithmic generality at the expense of empirical applicability. Although this paradigm enhances theoretical transferability, it constrains reproducibility and obscures performance under real-world conditions, where factors such as multi-tenant resource contention, network latency variability across availability zones, billing granularity, and service quotas critically shape system behavior (Nawrocki and Osypanka, 2021; Goyal et al., 2021; Zhou et al., 2021). This limitation becomes more pronounced when considering that no study validates its methods on dominant commercial providers such as AWS or Google Cloud Platform, despite their combined majority market share, while only a single study employs Azure ML Studio and another uses AWS without systematic cross-provider comparison (Nawrocki and Osypanka, 2021; Mayuranathan et al., 2022). Consequently, the absence of standardized validation undermines industry adoption and prevents the establishment of robust cross-platform benchmarks (Abdi et al., 2025; Golec et al., 2024).

A further structural weakness lies in the insufficient specification of cloud service models, as optimization strategies vary substantially across infrastructure-as-a-service (IaaS), platform-as-a-service (PaaS), and software-as-a-service (SaaS) environments. Most studies implicitly target IaaS contexts, focusing on virtual machine sizing, storage tiering, and network allocation (Abdi et al., 2025; Zhou et al., 2021; Rawat et al., 2020; Goyal et al., 2021; Kushwah and Ranga, 2021), while only limited attention is given to PaaS-level mechanisms such as container orchestration and serverless scaling (Nawrocki and Osypanka, 2021; Golec et al., 2024), and SaaS optimization remains entirely unaddressed. This imbalance is increasingly problematic as cloud computing shifts toward higher abstraction layers, including managed services and serverless architectures, where optimization constraints and performance levers differ fundamentally (Abdi et al., 2025; Zheng et al., 2020; Arul et al., 2023). Bridging this gap requires explicitly mapping research contributions to commercial ecosystems, where cost optimization approaches such as WOA-based methods can integrate with AWS Auto Scaling, Azure VM Scale Sets, or GCP instance groups (Goyal et al., 2021), security models such as deep learning-based intrusion detection systems can deploy through services like AWS SageMaker, Azure Machine Learning, or GCP Vertex AI (Hasimi et al., 2024; Sanjalawe and Althobaiti, 2023; Kushwah and Ranga, 2021), and resource allocation techniques can leverage monitoring infrastructures such as AWS CloudWatch, Azure Monitor, or GCP Cloud Monitoring (Zhou et al., 2021; Abdullah and Surputheen, 2024; Rawat et al., 2020). Similarly, hybrid and edge-oriented solutions align with platforms such as AWS Outposts, Azure Arc, AWS Wavelength, and Azure Edge Zones, reflecting the growing importance of distributed cloud-edge integration (Abdi et al., 2025; Zheng et al., 2020; Arul et al., 2023).

Addressing these limitations requires a systematic reorientation of research practices toward commercial relevance and methodological rigor. Future studies must prioritize validation on major cloud providers using realistic workloads and pricing schemes (Nawrocki and Osypanka, 2021; Mayuranathan et al., 2022), explicitly define the targeted service model and its associated constraints (Abdi et al., 2025; Zhou et al., 2021), and promote reproducibility through open-source implementations integrated with provider-specific software development kits (Goyal et al., 2021; Sanjalawe and Althobaiti, 2023). Moreover, strengthening industry–academia collaboration through research credit programmes can enable large-scale experimentation (Golec et al., 2024; Hasimi et al., 2024), while incorporating realistic billing mechanisms, including reserved instances and egress costs, can improve cost-model fidelity (Nawrocki and Osypanka, 2021; Rawat et al., 2020; Zhou et al., 2021). Finally, the development of standardized, cross-platform benchmark suites is essential to facilitate meaningful comparison and accelerate the translation of AI-driven optimization techniques into operational cloud environments (Abdi et al., 2025; Abdullah and Surputheen, 2024; Kushwah and Ranga, 2021).

### 5.2 Cloud platform considerations and commercial service alignment

The analysis of cloud platform distribution (Table 9) reveals a critical disconnect between academic research and industry practice in AI-driven cloud optimization. This section examines the implications of this gap and provides recommendations for aligning future research with commercial cloud service ecosystems.

#### 5.2.1 The platform-agnostic research paradigm

The finding that 66.7% of included studies propose platform-agnostic optimization approaches reflects a common pattern in academic research: prioritizing algorithmic generality over platform-specific implementation. While this approach has theoretical merit—ensuring that optimization principles are broadly applicable—it introduces several limitations in reproducibility, missed platform-specific opportunities, and validation constraints. Without empirical validation on commercial platforms, it is impossible to assess how proposed algorithms perform under real-world constraints such as multi-tenant resource contention, network latency variability across availability zones, platform-specific billing granularity, and service quotas and rate limits (Nawrocki and Osypanka, 2021; Goyal et al., 2021; Zhou et al., 2021).

#### 5.2.2 The AWS, azure, and GCP validation gap

Perhaps the most striking finding from Table 9 is that zero studies validated their approaches on AWS or Google Cloud Platform, despite these platforms collectively holding over 60% of the global cloud market share. Only (Nawrocki and Osypanka 2021) performed validation on Azure ML Studio, representing a mere 5.6% of included studies. Similarly, (Mayuranathan et al. 2022) employed AWS infrastructure for intrusion detection evaluation, yet without systematic comparison across providers. This validation gap has significant consequences for industry adoption, cross-platform comparison, and the establishment of standardized benchmarks for AI-driven optimization (Abdi et al., 2025; Golec et al., 2024).

#### 5.2.3 Service model implications: IaaS, PaaS, and SaaS

The ambiguity around cloud service models in the included studies represents another critical gap. AI-driven optimization strategies differ substantially across:

- **IaaS** (VM sizing, storage tiering, network bandwidth) (Abdi et al., 2025; Zhou et al., 2021; Rawat et al., 2020);
- **PaaS** (container orchestration, serverless scaling, and managed database performance) (Nawrocki and Osypanka, 2021; Golec et al., 2024);
- **SaaS** (user experience, API rate limiting, and multi-tenant allocation)—not covered by any included study.

The majority of included studies implicitly target IaaS environments (Zhou et al., 2021; Rawat et al., 2020; Goyal et al., 2021; Kushwah and Ranga, 2021), with limited attention to PaaS optimization (Nawrocki and Osypanka, 2021) and zero coverage of SaaS optimization. As cloud computing shifts toward higher abstraction levels (serverless, managed services, and SaaS), AI-driven optimization research has not kept pace (Abdi et al., 2025; Zheng et al., 2020; Arul et al., 2023).

#### 5.2.4 Connecting findings to commercial services

To bridge the research-practice gap, optimization findings from the included studies can be mapped to specific commercial services:

- **Cost optimization**—WOA-based approaches (Goyal et al., 2021) can be implemented via AWS Auto Scaling with custom metrics, Azure VM Scale Sets with the Cost Management API, or GCP Compute Engine instance groups via the Compute Engine API.
- **Security optimization**—deep learning-based IDS (Hasimi et al., 2024) can be deployed on AWS SageMaker with VPC Flow Logs, Azure Machine Learning with Network Watcher, or GCP Vertex AI with Cloud Audit Logs. Ensemble CNN–LSTM models (Sanjalawe and Althobaiti, 2023) and optimized ELM detectors (Kushwah and Ranga, 2021) follow the same deployment pattern.
- **Resource allocation**—differential evolution algorithms (Zhou et al., 2021) can be integrated with AWS CloudWatch, Azure Monitor, or GCP Cloud Monitoring to dynamically adjust cluster scaling parameters.
- **Hybrid and edge scenarios**—security-aware workflow scheduling (Abdi et al., 2025) maps naturally to AWS Outposts or Azure Arc, while edge-cloud resource management (Zheng et al., 2020; Arul et al., 2023) aligns with AWS Wavelength and Azure Edge Zones.

#### 5.2.5 Recommendations for future research

Based on the platform analysis (Table 9), future research should:

1. Validate proposed algorithms on at least one major commercial platform (AWS, Azure, or GCP) using real-world workloads and pricing models (Nawrocki and Osypanka, 2021; Mayuranathan et al., 2022).
2. Explicitly state which cloud service model (IaaS, PaaS, or SaaS) each optimization approach targets (Abdi et al., 2025; Zhou et al., 2021).
3. Release open-source implementations integrating with commercial cloud SDKs to facilitate reproducibility (Goyal et al., 2021; Sanjalawe and Althobaiti, 2023).
4. Leverage industry–academia partnership programmes (AWS Research Credits, Azure AI for Research, Google Cloud Research Credits) to enable large-scale empirical validation (Golec et al., 2024; Hasimi et al., 2024).
5. Incorporate realistic billing models including reserved instances, spot instances, and committed use discounts (Nawrocki and Osypanka, 2021; Rawat et al., 2020; Zhou et al., 2021).
6. Establish standardized cross-platform benchmarks enabling meaningful comparison across studies and providers (Abdi et al., 2025; Abdullah and Surputheen, 2024; Kushwah and Ranga, 2021).

### 5.3 Study limitations

Despite the methodological rigor applied through PRISMA 2020 and the PICOC framework, several limitations must be acknowledged. The review scope is restricted to studies published between 2020 and 2025, potentially excluding foundational earlier work. Heterogeneity in simulation environments, datasets, and reporting standards complicates direct metric comparisons and limits methodological standardization. The predominance of simulated scenarios rather than large-scale production deployments constrains generalization to industrial environments. Validation in multicloud or hybrid edge-cloud architectures remains limited across the reviewed literature. Several primary studies report performance metrics derived from single experimental runs without providing standard deviation or confidence intervals, preventing a robust assessment of statistical significance and potential variance overlap. The absence of a formal quantitative meta-analysis restricts the aggregated estimation of effect sizes. Finally, the reviewed studies exhibit limited coverage of VM migration dynamics and geographically distributed IaaS management, dimensions that are critical in practice and addressed by recent works not represented in the systematic review corpus. This gap reinforces the need for standardized benchmarking protocols across the field.

### 5.4 Recommendations for future research

The synthesized evidence enables the identification of specific priorities for future research. The development of standardized benchmarks and shared datasets—including realistic VM migration traces and geo-distributed workloads—is essential to enable homogeneous comparisons. Validation of optimization algorithms in real multicloud and edge-cloud deployments under high variability conditions is required to bridge the gap between simulation performance and production applicability. Integration of federated learning and distributed learning enables collaborative optimization while preserving data privacy across organizational boundaries. Comprehensive Total Cost of Ownership (TCO) evaluations that incorporate initial model training costs, maintenance overhead, and infrastructure transition costs are necessary for practitioner decision-making. Adoption of Explainable AI (XAI) frameworks will strengthen model interpretability and regulatory compliance in data-sensitive sectors. Finally, application of formal quantitative meta-analytical techniques will enable aggregated effect size estimation and improve the statistical robustness of future systematic reviews in this domain.

### 5.5 General implications

The results confirm that AI constitutes a structural mechanism for economic and operational optimization in cloud environments. The convergence across studies demonstrates that ML, deep learning, and metaheuristic techniques enable substantial cost reductions and significant improvements in resource efficiency. Nevertheless, methodological fragmentation and the lack of standardization indicate that the field remains in a consolidation phase. The development of autonomous, explainable, and distributed architectures—informed by emerging sustainability frameworks and VM migration models—represents the next stage in the evolution of intelligent cloud optimization.

In summary, this systematic review fulfills its stated objective by identifying, classifying, and critically analyzing AI models applied to cost and resource optimization in cloud computing, demonstrating their quantifiable impact and establishing a coherent agenda for future development.

## 6 Conclusions

The integration of AI and machine learning in cloud computing constitutes a structural mechanism for achieving measurable improvements in cost efficiency, energy management, resource utilization, and security. The synthesis of 18 primary studies, selected using the PRISMA 2020 protocol and PICOC framework, demonstrates that predictive provisioning, intelligent load balancing, metaheuristic optimization (e.g., Whale Optimization Algorithm and Particle Swarm Optimization), and deep learning-based intrusion detection systems collectively address persistent operational challenges in heterogeneous cloud environments. The magnitude of these benefits is contingent on architectural context, with more pronounced gains observed in elastic, pay-per-use infrastructures where economic incentives align with algorithmic efficiency—a dependency insufficiently articulated in prior fragmented reviews.

This review fulfills its objective by systematically identifying, classifying, and critically evaluating artificial intelligence techniques for cost and resource optimization. Dominant approaches include supervised learning for demand forecasting, metaheuristic algorithms for dynamic resource allocation, hybrid deep learning architectures combining convolutional and recurrent layers for threat detection, and Quality of Service-driven load balancing mechanisms. However, virtual machine migration dynamics and geographically distributed Infrastructure-as-a-Service management remain underrepresented, despite their practical relevance. Emerging contributions, including BackFillMe, FollowMe@LS, and energy-aware migration frameworks, have begun to address this gap.

Three limitations condition the interpretation of these findings. First, heterogeneity in simulation environments and datasets constrains direct cross-study comparability. Second, the predominance of simulated evaluations limits generalization to large-scale production systems. Third, inconsistent reporting of statistical dispersion, including the absence of standard deviation or confidence intervals in several studies, restricts the assessment of result robustness. These limitations do not undermine the consistency of observed trends but reduce the precision of aggregate performance claims.

Future research should prioritize five directions. First, the development of standardized benchmarks that incorporate virtual machine migration traces and geographically distributed workloads. Second, empirical validation in real multicloud and edge-cloud environments under dynamic demand conditions. Third, the integration of federated and distributed learning for privacy-preserving optimization across organizational boundaries. Fourth, the adoption of explainable artificial intelligence frameworks to enhance interpretability and support regulatory compliance in sensitive domains. Fifth, the application of formal quantitative meta-analysis to enable statistically grounded aggregation of effect sizes. Collectively, these directions define a pathway toward autonomous, interpretable, and sustainable intelligent cloud systems.

## Data Availability

The original contributions presented in the study are included in the article/supplementary material, further inquiries can be directed to the corresponding author.
